# Supplementary material for: MetaRibo-Seq measures translation in microbiomes
Source: Nat Commun. 2020 Jun 29;11:3268. doi: 10.1038/s41467-020-17081-z (PMC7324362; doi:10.1038/s41467-020-17081-z)
Supplement: Supplementary file 10 — Supplementary Data 7 [file 41467_2020_17081_MOESM10_ESM.zip › File2/Confidence_High_Taxonomy/402994_out.krona.html]

Javascript must be enabled to view this page.

members
magnitude
magnitudeUnassigned
count
unassigned
taxon
rank

402994\_out

34

2
34
superkingdom

34
phylum
976

34
200643
class

34
order
171549

family
34
815

genus
34
1

SRS043701\_contig\_number\_9363
816


SRS142712\_contig\_number\_900
species
1
28116

species
1
2292923

SRS016381\_contig\_number\_contig-100\_32.34870

1
2292941
species

SRS013215\_contig\_number\_455


SRS043001\_contig\_number\_1823
1
371601
species


SRS014613\_contig\_number\_3385
species
1
2292935


SRS013476\_contig\_number\_20835SRS013940\_contig\_number\_10168SRS014923\_contig\_number\_27403SRS016018\_contig\_number\_1512SRS018575\_contig\_number\_5462SRS024132\_contig\_number\_8415SRS024388\_contig\_number\_339SRS024549\_contig\_number\_4825SRS048060\_contig\_number\_3774SRS064232\_contig\_number\_1420SRS075341\_contig\_number\_13394SRS077024\_contig\_number\_9379SRS098655\_contig\_number\_8665SRS098717\_contig\_number\_484SRS1041145\_contig\_number\_31909SRS104636\_contig\_number\_15451SRS149784\_contig\_number\_25830
17
671267
species


SRS1041137\_contig\_number\_27685
1
47678
species

1
species
246787

SRS058070\_contig\_number\_1496

2
species
821

SRS017103\_contig\_number\_16159SRS045826\_contig\_number\_1657


SRS019286\_contig\_number\_3806SRS023346\_contig\_number\_2472SRS042628\_contig\_number\_5438SRS054590\_contig\_number\_606SRS057717\_contig\_number\_5746SRS1041038\_contig\_number\_16488SRS140645\_contig\_number\_690
7
357276
species
